# “Wolf Shifts” and Their Physical Interpretation Under Laboratory Conditions

**DOI:** 10.6028/jres.098.018

**Published:** 1993

**Authors:** Klaus D. Mielenz

**Affiliations:** Alpine Lake Resort, Terra Alta, WV 26764-0402

**Keywords:** classical optics, coherence theory, conservation of energy, correlation-induced spectral changes, diffraction, interference, propagation of light, radiometry, spectral invariance, spectral preservation, spectroscopy, Wolf shifts

## Abstract

This paper attempts to reconcile conflicting points of view of laboratory physicists and coherence theorists on correlation-induced spectral changes arising from the partial coherence of primary and secondary light sources. It is shown that, under normal laboratory conditions and in the Fraunhofer approximation, the directional spectrum of light does not change on propagation in free space, and that each frequency component of the total spectrum is preserved in accordance with the principle of energy conservation. It is demonstrated, and illustrated by examples, that descriptions of diffraction by the theory of partial coherence and by classical wave optics are fully equivalent for incoherent primary sources. A statistical approach is essential, and coherence theory is required, for partially coherent primary sources.

## 1. Introduction

“The number of different optical phenomena has become in our time so great that caution must be taken so as to avoid being deceived, and also to refer the phenomena always to the simple laws. This is more necessary in the case of diffraction, as we shall see, than in all the other phenomena.” This quote appeared in a classical memoir, “New Modification of Light by the Mutual Influence and the Diffraction of the Rays,” which Fraunhofer published in 1821 [1]. It is still apropos today, as new issues concerning the modification of spectra by diffraction have arisen in the recent past.

In a paper published in 1986, Wolf [2] raised the interesting question “whether the normalized spectrum of light remains unchanged on propagation through free space.” He considered this to be the case when the spectrum from a partially coherent source is emitted isotropically. He coined the term “invariance of the spectrum of light on propagation” to describe this isotropic propagation of a spectrum and noted that obviously there exist sources whose spectra are not emitted isotropically. The spectral properties of such sources have subsequently become known as “correlation-induced spectral modifications” or “Wolf shifts.” In this context, the word “source” denotes either a “primary” (physical) source of radiation, or a “secondary” source such as a diffracting aperture.

Wolf has emphasized that he was concerned with physical mechanisms that produce spectral modifications of a still unknown nature. For example, in Ref. [2] he mentioned astrophysical measurements and posed the question “whether source correlations may perhaps not give rise to differences between the spectrum of the emitted light and the spectrum of the detected light that originates in some stellar sources.” In Ref. [3] he considered non-cosmological source correlations which “must clearly be manifestations of some cooperative phenomena. At the atomic level possible candidates may perhaps be superradiance and superfluorescence.”

Wolf’s papers prompted a large number of publications by himself and others on the subject of correlation-induced modifications. Regrettably, this literature includes papers which have led to misinterpretations of the physical nature of Wolf shifts. For the most part, these papers analyzed classical diffraction and interference experiments with incoherent sources within the context of coherence theory. It is likely that they were merely intended to convey illustrations of correlation-induced modifications, but nonetheless they have raised questions concerning the similarity and differences between statistical optics and classical wave optics. The present paper attempts to clarify these issues insofar as laboratory applications of optical theories are concerned.

## 2. Outline

There are three major questions that have been raised with respect to Wolf shifts encountered in the laboratory.

*Does the spectrum of partially coherent light change on propagation in free space, and are such changes consistent with the principle of energy conservation?* This question arises from repeated statements, e.g., Refs. [3–7] that the spectrum of light is not invariant on free-space propagation. For example: “It has been demonstrated in the last few years, both theoretically and experimentally that, in general, the spectrum of light generated by a partially coherent source changes on propagation, even in free space” [5]. The experiments cited here did not pertain to free-space propagation in a literal sense but were diffraction [8–10] or interference [11] experiments in which the spectra observed in different directions were found to be different, as might be expected in such experiments. In Sec. 4 of this paper, we address the issue of energy conservation from the point of view that “free-space propagation” means the unimpeded propagation of light outside of sources and in the absence of absorption, photoluminescence and similar mechanisms that can destroy or create spectral components. Diffraction at clear apertures is not regarded as free-space propagation, since apertures impede incident wave fronts by truncating them. The word “spectrum” is interpreted as the functional form of the spectral concentrations of measurable radiant quantities. We distinguish between “directional spectra” which are observed in a given direction, and “total spectra” which are obtained by (physical or mathematical) integrations of spectral radiant quantities over a specified space domain. A directional spectrum is considered “unchanged” if the relative spectral distribution of the pertinent spectral concentration does not change along the path of a ray. A total spectrum is considered “preserved” if it does not change within the boundaries of the specified domain. Our conclusion will be that coherence theory does *not* predict spectral modifications of directional spectra due to the free-space propagation of light in normal laboratory situations, and that total spectra obey the energy principle for each spectral component separately.

*Do the theory of partial coherence and the classical Huyghens-Fresnel-Kirchhoff diffraction theory give different results in situations that involve incoherent physical sources? Which of them should be applied for solutions of practical problems?* These questions arise from publications in which Fraunhofer diffraction [12] and Young’s interference experiment [13,14] were reexamined by coherence theory for incoherent primary sources. The findings of these publications were consistent with classical results, but were presented in a manner so highly abstract that they could be mistaken as manifestations of hitherto unknown phenomena. In Sec. 5 of this paper, we will demonstrate that the theory of partial coherence and the Huyghens-Fresnel-Kirchhoff theory give identical results in the case of an incoherent primary source. We will emphasize that coherence theory is required if the primary source is partially coherent.

*Are traditional radiometric practices afflicted by previously unknown errors due to the partial coherence of light?* One of the above-mentioned papers [9] reported experimental results that conflicted with classical diffraction theory for incoherent primary sources such as those used, but was repeatedly cited [6,9,12] as evidence that radiometric measurements by national standardizing laboratories may be in error due to a lack of consideration of Wolf shifts. For example: “… the large scatter which exists in the spectroradiometry scales maintained by different national laboratories has up to now not been satisfactorily explained. … The spectrum of the transmitted radiation undergoes frequency shifts … which were not taken into account in specifying the spectroradiometric scales” [6]. Nugent and Gardner [15] have since shown that, for an incoherent source and an optical system consisting of a series of thin apertures and lenses, diffraction is the only physical effect that can introduce spectral changes, and that diffraction effects are too small to be a significant source of error in spectroradiometric calibrations. As their paper has effectively addressed the issue of calibration errors, this question will not be pursued further in this paper except within the context of an example given in Sec. 5.2. However, most of the conclusions of the paper are pertinent to optical radiometry with partially coherent physical sources.

In Sec. 3, we define relevant terms which could otherwise be misunderstood or interpreted differently. Section 6 contains concluding remarks.

## 3. Definitions

**Fig. 1:**
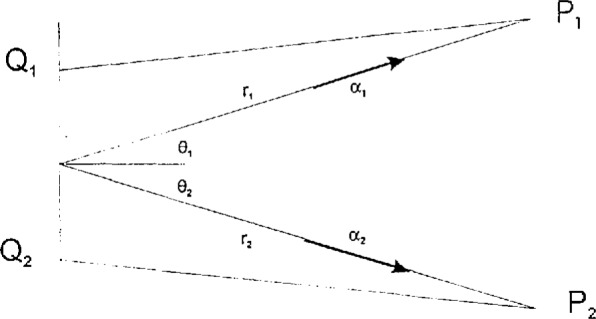
Notation pertaining to free-space propagation of cross-spectral density. The points and light paths shown are not coplanar.

To avoid the risk of misunderstandings due to poorly defined nomenclature, the most important terms and symbols used in this paper are summarized in the following. For a more detailed explanation of these terms, the reader is referred to pertinent review articles [16–19] and textbooks [20–22]. The following symbols will be used (see Figs. 1, 3, 5):

*t* denotes time, *τ* is a time delay, *c* is the speed of light in vacuum, λ is the wavelength, ω = 2π*c*/λ is the circular frequency, and *k* = 2π/λ = ω/*c* is the circular wavenumber of the light;

*P* and *Q* are points in space, ***P*** and ***Q*** are their position vectors with respect to the origin of a coordinate system, d***P*** and d***Q*** are area elements at these points, *PQ* is the distance and ***PQ*** is the vector from *P* to *Q*, ***n*** is a surface normal and (***n***,***PQ***) is the angle enclosed by this normal and the vector ***PQ***.

Additional symbols will be introduced as needed. Lightface type is used for scalar quantities, and boldface for vectors and area elements. Multiple integral signs are avoided where possible so that, for example, ∫d***P***d***Q*** … denotes a four-fold integral.

The basic quantities of coherence theory used in this paper are the “mutual coherence function” *Γ*(*P*_1_, *P*_2_, τ) of two points in an optical radiation field (the point *P*_1_ being considered at a time τ later than the point *P*_2_) and its Fourier transform, the “cross-spectral density” *W*(*P*_1_,*P*_2_,ω):

$$\begin{array}{c} \Gamma \left(P_{1},P_{2},\tau \right)=<V\left(P_{1},t+\tau \right)V*\left(P_{2},t\right)>\\ =\int d\omega \;e^{-i\omega \tau }W\left(P_{1},P_{2},\omega \right), \end{array}$$ (1)

$$W\left(P_{1},P_{2},\omega \right)=\left(1/2\pi \right)\int d\tau \;e^{i\omega \tau }\Gamma \left(P_{1},P_{2},\tau \right),$$ (2)

where *V*(*P_i_,t*) is the complex wave amplitude (as defined in coherence theory) of the radiation field at the space-time point (*P_i_,t*)V* is the complex conjugate, and <…> denotes a finite power signal or an ergodic ensemble average. The cross-spectral density *W*(*P*_1_,*P*_2_,*ω*) has no negative-frequency components.

The following special forms of these functions are required for physical interpretations of theoretical results: The radiant flux density at a surface element d***P***_⊥_ perpendicular to the direction of light propagation at the point *P*,

$$E\left(P\right)=\Gamma \left(P,P,0\right)=d\Phi /dP_{⊥}\left[N/m^{2}\right],$$ (3)

and the spectral radiant flux density (spectral concentration of *E*_ω_(*P*) with respect to frequency) at *P*,

$$\begin{array}{c} E_{\omega }(P)=W\left(P,P,\omega \right)=d^{2}\Phi /\left(dP_{⊥}d\omega \right)\\ =d\Phi_{\omega }/dP_{⊥}\left[{\text{N/m}}^{2}/\text{Hz}\right], \end{array}$$ (4)

where *Φ* is the radiant flux, and d*Φ_ω_* is the spectral radiant flux incident on d**P**_⊥_. Radiant flux, radiant flux density, and spectral radiant flux density are often called “energy flux,” “optical intensity” and “spectral power density,” and are denoted by different symbols in theoretical optics. In this paper, the terms and symbols recommended in the *International Lighting Vocabulary* [23] will be used.

Spectral concentrations with respect to frequency, wavelength, etc., are denoted by subscripts, ω, λ, etc., and are also referred to by the adjective “spectral.” The term “normalized spectrum” introduced by Wolf [2] refers to the spectral concentration of a radiant quantity divided by that quantity itself, so that the ratio *Φ*_ω_/*Φ* represents the normalized frequency spectrum of radiant flux. This normalization appears to have no effect on the topics discussed in this paper.

The quantities defined by Eqs. (1) through (4) are often evaluated in a “far-field” approximation. In most papers on coherence this appears to be the Fraunhofer approximation of classical diffraction theory. To avoid confusion, we will specify all approximations made in this paper.

## 4. Free-Space Propagation of Cross-Spectral Density. Spectral Preservation

The propagation of cross-spectral density in free space is governed by two Helmholtz equations,

$$\begin{array}{cc} \left(\Delta_{i}+k^{2}\right)W\left(P_{1},P_{2},\omega \right)=0, & i=1,2, \end{array}$$ (5)

which can be solved by means of Green functions in a manner analogous to that used in Kirchhoff’s diffraction theory. According to Parrent [24], the general solution for the cross-spectral density at two space points *P*_1_ and *P*_2_, due to a plane polychromatic source, is

$$\begin{array}{l} \;W\left(P_{1},P_{2},\omega \right)=1/{\left(2\pi \right)}^{2}\int dQ_{1}dQ_{2}\\ \;\times \cos\left(n,Q_{1},P_{1}\right)\cos\left(n,Q_{2}P_{2}\right)\\ \times \left[\left(1-ikQ_{1}P_{1}\right)\left(1+ikQ_{2}P_{2}\right)/{\left(Q_{1}P_{1}Q_{2}P_{2}\right)}^{2}\right]\\ \times W\left(Q_{1},Q_{2},\omega \right)\exp\left[ik\left(Q_{1}P_{1}-Q_{2}P_{2}\right)\right], \end{array}$$ (6)

where *Q*_1_ and *Q*_2_ are two points inside the source domain and the other symbols are explained in Fig. 1. This equation is usually simplified by making two geometrical assumptions:

1. The distances *Q*_1_*P*_1_ and *Q*_2_*P*_2_ are large compared to the wavelength λ, so that
  $$\begin{array}{c} \left(1-ikQ_{1}P_{1}\right)\left(1+ikQ_{2}P_{2}\right)/{\left(Q_{1}P_{1}Q_{2}P_{2}\right)}^{2}\\ ∼k^{2}/\left(Q_{1}P_{1}Q_{2}P_{2}\right). \end{array}$$ (7)
2. The angles (***n,Q***_1_***P***_1_) and (***n,Q***_2_***P***_2_) and the factor 1/(*Q*_1_*P*_1_ *Q*_2_*P*_2_) do not vary appreciably within the source domain and are approximated by their values at the source center, so that
  $$\begin{array}{c} \cos\left(n,Q_{1}P_{1}\right)\cos\left(n,Q_{2}P_{2}\right)/\left(Q_{1}P_{1}Q_{2}P_{2}\right)\\ ∼\cos\theta_{1}\cos\theta_{2}/\left(r_{1}r_{2}\right). \end{array}$$ (8)
  This gives
  $$\begin{array}{c} W\left(P_{1},P_{2},\omega \right)=\left(\cos\theta_{1}\cos\theta_{2}\right)/\left(\lambda^{2}r_{1}r_{2}\right)\\ \times \int dQ_{1}dQ_{2}W\left(Q_{1},Q_{2},\omega \right)\exp\left[ik\left(Q_{1}P_{1}-Q_{2}P_{2}\right)\right]. \end{array}$$ (9)

In the Fraunhofer approximation, the distances *Q*_1_*P*_1_ and *Q*_2_*P*_2_ in the argument of the exponential function are expressed by

$${\left(Q_{i}P_{i}\right)}^{2}={\left(r_{i}\alpha_{i}-Q_{i}\right)}^{2},\;Q_{i}P_{i}\sim r_{i}-\alpha_{i}\cdot Q_{i}.$$ (10)

Hence the spectral radiant flux d*Φ*_ω_ defined by Eq. (4) may be evaluated by substituting *P*_1_*=P*_2_*=P, r*_1_*=r*_2_*=r, θ*_1_=θ_2_=0, and ***α***_1_ = ***α***_2_ = ***α*** into Eq. (9). Thus,

$$\begin{array}{l} d\Phi_{\omega }=dP_{⊥}W\left(P,P,\omega \right)∼\cos^{2}\theta \sin\theta \;d\theta \;d\phi \\ \times \int dQ_{1}dQ_{2}W\left(Q_{1},Q_{2},\omega \right)\exp\left[-ik\alpha •\left(Q_{1}-Q_{2}\right)\right], \end{array}$$ (11)

where d***P***_⊥_=*r*^2^sin*θ*d*θ*d*ϕ*, and ***α*** = (cos*ϕ*sin*θ*, sin*ϕ*sin*θ*,cos*θ*) is the unit vector in the direction of light propagation at *P.* This shows that d*Φ_ω_* depends only on the angular position of the point of observation *P*, not on its distance from the source. Therefore, the directional radiant flux spectrum does not change on propagation in free space.

The total radiant flux spectrum emitted by the source into the positive hemisphere is obtained by integrating Eq. (11) over all directions: 0 ≤ *θ* ≤ π/2 and 0 ≤ *ϕ* ≤ 2π. Introducing sum and difference coordinates in the source plane,

$$\begin{array}{cc} Q_{+}=\frac{1}{2}\left(Q_{1}+Q_{2}\right), & Q_{-}=Q_{1}-Q_{2}, \end{array}$$ (12)

and reversing the order of integrations, we find

$$\begin{array}{l} \Phi_{\omega }=\int d\Phi_{\omega }∼\int dQ_{+}\{\int dQ_{-}W\left(Q_{+}+\frac{1}{2}Q_{-},Q_{+}-\frac{1}{2}Q_{-},\omega \right)\\ \;\times \int d\theta \sin\theta \cos^{2}\theta \int d\phi \;\exp\left(-ik\alpha \cdot Q_{-}\right)\}. \end{array}$$ (13)

The triple integral in curled brackets that appears in this expression depends on ***Q***_+_ only. Therefore, Eq. (13) shows that the radiant flux spectrum, integrated over all directions, is equal to a single integral over the source plane. This implies preservation of the spectrum, as defined in Sec. 2. Equation (13) can be brought into a form more familiar to radiometrists,

$$\Phi_{\omega }=\int dQ_{+}M_{\omega }\left(Q_{+}\right),$$ (14)

by defining

$$\begin{array}{c} M_{\omega }\left(Q_{+}\right)=\left\{\ldots \right\}=\left(2\pi /\lambda^{2}\right)\int dQ_{-}\\ \times W\left(Q_{+}+\frac{1}{2}Q_{-},Q_{+}-\frac{1}{2}Q_{-},\omega \right)j_{1}\left(k\left|Q_{-}\right|\right)/\left(k\left|Q_{-}\right|\right) \end{array}$$ (15)

as the “generalized spectral radiant exitance” of the source. Equation (15), where *j*_1_ is the spherical Bessel function of the first kind and order, was derived by Marchand and Wolf [16].

**Fig. 2:**
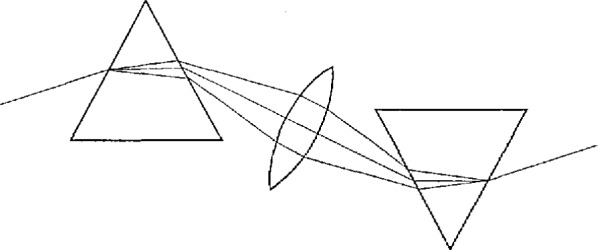
Newton’s *experimentum crucis* on spectral preservation.

Equations (11) and (13) constitute the laws of energy conservation, in the Fraunhofer approximation, for the free-space propagation of light from a planar source of any coherence. Equation (11) states that the directional spectrum does not change on propagation in free-space. Equation (13) states that energy is conserved for each spectral component separately. If the light propagation is anisotropic, the directional radiant flux spectrum will generally differ from the radiant exitance spectrum, but spectral components missing in any one direction of propagation will be present in other directions. This is reminiscent of Newton’s famous *experimentum crucis* depicted in Fig. 2, which proved that the colors dispersed by one prism can be recombined by another. It is not difficult to imagine similar ways of recovering a source spectrum that propagates anisotropically. For example, if the “source” is a diffracting aperture illuminated by an incoherent physical source, then, the diffracted light will be spectrally anisotropic. It can, however, be collected by an integrating sphere so that under idealized conditions the spectral radiant exitance of the exit port of the sphere will be an exact duplicate of the spectral radiant exitance of the physical source. Under these circumstances, the total spectrum has been “preserved.”

Wolf [2] expressed the belief that the tacit assumption of isotropy of spectral emission is “implicit in all of spectroscopy [but] does not appear to have been previously questioned because with light from traditional sources one has never encountered any problems with it.” A different point of view is adopted in this paper. The isotropic propagation of a source spectrum may perhaps be an assumption dictated by necessity in astrophysical observations when it is not possible to study the light from stellar objects in different directions. However, this assumption would hardly be made tacitly by experienced spectrometrists dealing with man-made sources. There are probably no laboratory sources at all that emit strictly isotropic radiation. Physical realizations of blackbody sources incorporate apertures that diffract the isotropic thermal radiation generated inside the blackbody cavity, before it emerges into free space. The Planckian emission of metal-strip or coil lamps is modified by the spectral emissivity of the metal, which depends on wavelength as well as direction and can therefore cause spectrally dependent anisotropics. Additionally the finite size of the emitting strip or coil introduces diffraction and polarization effects, and coil lamps exhibit shadowing effects that cause a directionality of emission. In absorption spectrometry, spectral anisotropy can arise from the dichroism or pleochroism of samples. In photoluminescence spectrometry, the spectral anisotropy of polarized samples is a commonly known phenomenon. Accordingly, carefully performed spectrometric experiments usually involve a “mapping” of the radiation emitted or transmitted by sources and samples. That is, directional and spectral scans are performed to assess the magnitude of departures from the idealized case of angular and spectral isotropy. The tacit assumption that is made when such measurements are performed is, not that the spectrum is invariant in the sense defined by Wolf, but that it is preserved in the sense of Eq. (13).

Wolf and Gamliel [7] have cited an equation similar to Eq. (14) and, likewise, considered it to be a manifestation of energy conservation for each frequency component. However, they asserted that “there is no contradiction with the energy conservation law when the normalized spectrum changes on propagation.” Their argument appears to be that generalized source quantities such as the spectral radiant exitance of Eq. (15) are defined in terms of the Fraunhofer approximation of the cross-spectral density *W*(*P*_1_,*P*_2_,*ω*), and that this approximation is not valid in the source plane. This is true. The evaluation of diffraction integrals in close proximity to a physical source is difficult and has been attempted only in special cases. For example, Agrawal and Gamliel [4] have presented theoretical results for the paraxial propagation of partially coherent light at distances on the order of a few hundred wavelengths from a planar source. In one case considered they found that the spectrum, viewed at a fixed angle, first shifts towards the blue and then towards the red as the propagation distance increases. It is not within the purview of this paper to discuss these findings; the authors used a different mathematical approach and did not quantify the accuracy of their calculations. On the other hand, it should be noted that laboratory determinations of source parameters always involve measurements made at a distance. To determine the spectral radiant exitance of Eq. (15), a spectrora-diometer would be used to measure the spectral radiant flux of Eq. (11) for specified geometrical conditions, and often the measurement would be performed under conditions that justify the Fraunhofer approximation. Thus, Eqs. (13) and (15) may suffice to demonstrate the invariance of light propagation in free space and spectral preservation under normal laboratory conditions. Because of various approximations made in deriving propagation equations such as Eq. (6), it may not even be possible at all to rigorously “prove” energy conservation. On the other hand, it seems safe to presume that conservation of energy, if not assumed explicitly in coherence theory, is implicit in the electromagnetic theory underlying it. The Helmholtz equations [Eq. (5)] apply to individual frequencies, and thus appear to imply that spectra do not change on free-space propagation and are preserved.

## 5. Similarities and Differences Between Coherence Theory and Classical Wave Optics

### 5.1 Propagation of Cross Spectral Density and the Huyghens-Fresnel-Kirchhoff Principle

Turning to the question how spectral modifications due to partial coherence differ from classical diffraction and interference effects, we note that the Helmholtz equations [Eq. (5)] for the free-space propagation of cross-spectral density were derived from the corresponding Helmholtz equation for classical wave amplitudes [24]. This suggests that Eq. (6) is, in fact, a straightforward generalization of classical diffraction theory.

**Fig. 3:**
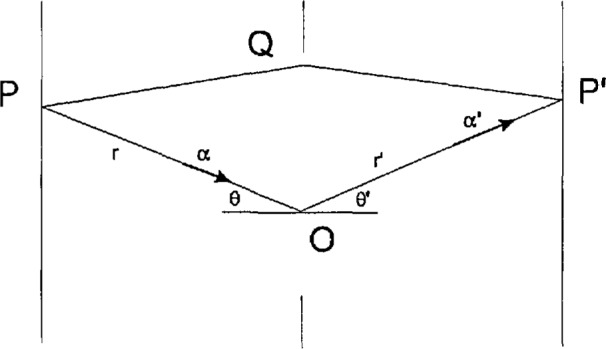
Notation pertaining to diffraction at an aperture. The points and light paths shown are not coplanar. The points *P*, *Q*, and *P*′ represent either of the points *P*_1_, *Q*_1_, and *P*′_1_ mentioned in Sec. 5.1.

There is, however, a practical difference in that Eq. (6) traces the propagation of cross-spectral density only from one pair of points to another, while the classical Huyghens-Fresnel-Kirchhoff diffraction integral traces the propagation of wave amplitudes from a source to an aperture and on to a point of observation in one sweep (Fig. 3). In order to eliminate this difference, we can apply Eq. (6) twice; first, from a pair of source points (*P*_1_,*P*_2_) to a pair of points (*Q*_1_,*Q*_2_) in the aperture plane,

$$\begin{array}{c} W\left(Q_{1},Q_{2},\omega \right)=\left(\cos\theta_{1}\cos\theta_{2}\right)/\left(\lambda^{2}r_{1}r_{2}\right)\\ \times \int dP_{1}dP_{2}W\left(P_{1},P_{2},\omega \right)\;\exp\left[ik\left(P_{1}Q_{1}-P_{2}Q_{2}\right)\right]; \end{array}$$ (16)

and then from (*Q*_1_,*Q*_2_) to a pair of points (*P*′_1_,*P*′_2_) in the diffracted wave field,

$$\begin{array}{c} W\left({P^{\prime }}_{1},{P^{\prime }}_{2},\omega \right)=\left(\cos{\theta^{\prime }}_{1}\cos{\theta^{\prime }}_{2}\right)/\left(\lambda^{2}{r^{\prime }}_{1}{r^{\prime }}_{2}\right)\times \\ \int dQ_{1}dQ_{2}W\left(Q_{1},Q_{2},\omega \right)\times \exp\left[ik\left(Q_{1}{P^{\prime }}_{1}-Q_{2}{P^{\prime }}_{2}\right)\right]. \end{array}$$ (17)

Substituting the first equation into the second, we find

$$\begin{array}{l} \;W\left({P^{\prime }}_{1},{P^{\prime }}_{2},\omega \right)=\left(1/\lambda^{4}\right)\left(\cos{\theta^{\prime }}_{1}\cos{\theta^{\prime }}_{2}/{r^{\prime }}_{1}{r^{\prime }}_{2}\right)\\ \;\times \int dP_{1}dP_{2}\left(\cos\theta_{1}\cos\theta_{2}/r_{1}r_{2}\right)W\left(P_{1},P_{2},\omega \right)\\ \times \int dQ_{1}dQ_{2}\exp\left[ik\left(P_{1}Q_{1}+Q_{1}{P^{\prime }}_{1}-P_{2}Q_{2}-Q_{2}{P^{\prime }}_{2}\right)\right]. \end{array}$$ (18)

Therefore, from Eq. (4) for *P'*_1_*=P'*_2_*=P'*, *r'*_1_*=r'*_2_*=r'*, *θ'*_1_ *= θ'*_2_*= θ'*,

$$\begin{array}{l} \;E_{\omega }\left(P^{\prime }\right)=W\left(P^{\prime },P^{\prime },\omega \right)=\left(1/\lambda^{4}\right)\left(\cos^{2}\theta^{\prime }/{r^{\prime }}^{2}\right)\\ \;\times \int dP_{1}dP_{2}\left(\cos\theta_{1}\cos\theta_{2}/r_{1}r_{2}\right)W\left(P_{1},P_{2},\omega \right)\\ \times \int dQ_{1}dQ_{2}\exp\left[ik\left(P_{1}Q_{1}+Q_{1}P^{\prime }-P_{2}Q_{2}-Q_{2}P^{\prime }\right)\right]. \end{array}$$ (19)

These equations are valid in the geometrical approximations of Eqs. (7) and (8), without approximations made with respect to the argument of the exponential functions in the integrands.

If the primary source is planar and incoherent, Eq. (19) can be simplified further by expressing the cross-spectral density inside the first integral by an expression given by Marchand and Wolf [16],

$$W\left(P_{1},P_{2},\omega \right)=\left(\lambda^{2}/\cos\theta_{1}\right)L_{\omega }\left(P_{1},\alpha \right)\delta \left(P_{2}-P_{1}\right),$$ (20)

where *L*_ω_(*P*_1_,***α***) is the spectral radiance at the source point *P*_1_ and in a direction ***α***, and δ is a delta function. Letting *P*_1_ *= P, r*_1_ *= r, θ*_1_ = *θ*, we obtain

$$\begin{array}{l} E_{\omega }\left({P^{\prime }}_{1}\right)=\left(1/\lambda^{2}\right)\left(\cos^{2}\theta^{\prime }/{r^{\prime }}^{2}\right)\int \left(dP\cos\theta /r^{2}\right)L_{\omega }\left(P,\alpha \right)\\ \;\times \int dQ_{1}dQ_{2}\exp\left[ik\left(PQ_{1}+Q_{1}P^{\prime }-PQ_{2}-Q_{2}P^{\prime }\right)\right]\\ \;=\left(1/\lambda^{2}\right)\left(\cos^{2}\theta^{\prime }/{r^{\prime }}^{2}\right)\int d\Omega L_{\omega }\left(P,\alpha \right)|\\ \;\times \int dQ\exp{\left[ik\left(PQ+QP\right)\right]|}^{2}, \end{array}$$ (21)

where d*Ω* is the solid angle element subtended at the aperture center *O* by a source area element at *P*, and ***α*** is the unit vector in the direction *PO.*

Since Eq. (21) can be recognized as the classical Huyghens-Fresnel-Kirchhoff diffraction formula for an extended incoherent source, we may conclude:

Classical optics and the theory of partial coherence give precisely the same results when applied to diffraction problems that involve an incoherent primary source. It is immaterial which approach is used in practice. Laboratory physicists appear to prefer the Huyghens-Fresnel-Kirchhoff formula, perhaps because they are more familiar with it or because the partial coherence of the radiation incident upon the aperture is already built into the formula. Coherence theorists apparently prefer to think of apertures as secondary sources, and use the van Cittert-Zernicke theorem to describe their coherence properties. The results are the same.

Classical diffraction theory is not applicable if the primary source is partially coherent. In this case, a statistical treatment is required and coherence theory must be used. Simple optical systems, such as a single aperture or lens, can be analyzed by single formulae such as Eqs. (18) and (19). However, for more complicated systems involving multiple apertures or lenses, a stepwise approach may be more practicable because multiple combinations of Eq. (6) quickly lead to unwieldy expressions.

It should be noted that the equations of this section no longer describe the free-space propagation of light and, therefore, no longer imply spectral preservation. There are two cases to be distinguished when light is diffracted at an aperture:

1. If the incident light is spectrally isotropic, the spectral concentration of radiant flux will be uniform inside the aperture and the total spectrum of the diffracted light will differ from the total source spectrum only by a constant (frequency-independent) factor. The *relative* (“normalized”) source spectrum will be preserved.
2. If the incident is spectrally anisotropic, the truncation of wavefronts at the aperture will be spectrally selective. The total spectrum of the diffracted light will be different from the source spectrum by a frequency-dependent factor. The *absolute* and *relative* source spectra will *not* be preserved.

These cases occur irrespective of the state of coherence of the primary physical source. Because spectral isotropy has generally been attributed to incoherent sources only, an important result of Wolf’s original paper [2] is that he has postulated the existence of partially coherent isotropic sources whose relative spectra will also be preserved upon diffraction, and that he has rigorously defined the properties of such sources. This is likely to have a significant impact on laboratory spectrometry. For example, the development of new imaging devices [8,11] which obey the conditions formulated by Wolf may be expected to reduce diffraction errors in optical radiometry.

### 5.2 Example: Axial Diffraction Loss Due to a Circular Aperture

The first assertion that “the large scatter found in the intercomparison of spectroradiometric scales is attributed to the spectral shifts” may be found in the above-referenced paper by Kandpal, Vaisha, and Joshi [9]. These authors stated that “apertures make the incoherent light partially coherent and a source correlation is introduced which along with the optics involved violates certain scaling law thereby modifying the spectral distribution of the source in the far zone.”

**Fig. 4:**
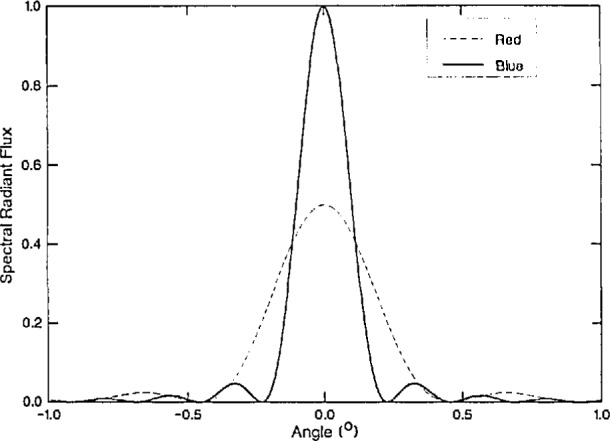
Diffraction patterns for red and blue light.

Diffraction errors in radiometry are, of course, well understood [25–34] by laboratory physicists. The phenomenological interpretation of these errors is that, on account of the scale factor 1/λ^2^ in the Huyghens-Fresnel diffraction integral Eq. (21), the different spectral components of light incident on an aperture are diffracted differently. If observations are made on axis the spectral radiant flux will be found to be “blue-shifted,” because diffraction at an aperture spreads red light farther into the off-axis region than blue light (Fig. 4). For a circular aperture illuminated by a point source this is described, in the Fraunhofer approximation, by the Airy diffraction formula found in textbooks of optics. By reciprocity, the same treatment applies for an extended, incoherent circular source and a point detector on axis [26]. The result is a classical formula which was originally derived by Rayleigh [35],

$$\epsilon \left(0\right)=J_{0}^{2}\left(kaw\right)+J_{1}^{2}\left(kaw\right),$$ (22)

where *ϵ*(0) is the axial diffraction loss, *J*_0_ and *J*_1_ are Bessel functions of the first kind, *a* is the radius of the aperture, and *w* is the angular radius of the source.

Foley [12] used the theory of partial coherence to derive the same result in a different form,

$$\begin{array}{c} S\left(z,\omega \right)={\left(af/a_{s}z\right)}^{2}S^{\left(0\right)}\left(\omega \right)\{1-J_{0}^{2}\left[3.832a/L\left(\omega \right)\right]\\ -J_{1}^{2}\left[3.832a/L\left(\omega \right)\right]\}, \end{array}$$ (23)

where *S*(*z*,*ω*) is the axial “spectrum” observed at a distance *z* from the aperture, *a* and *a_s_* are the respective radii of the aperture and source, *f* is the focal length of a lens illuminating the aperture, *S*^(0)^(*ω*) is the “source spectrum” and *L*(*ω*) = 3.832*f*/*ka*_s_. Foley’s explanation of Eq. (23) was “that the spectrum of the light at an on-axis observation point in the far zone differs from the spectrum in the aperture by a simple geometrical factor and a frequency dependent factor … that depends only on the ratio … of the aperture to the effective correlation length of the light in the aperture at frequency *ω.* Hence the difference between the spectrum of the light at an on-axis observation point in the far zone and the spectrum of the light in the aperture is determined by whether the light in the aperture is effectively spatially coherent over it, i.e. whether the light in the aperture is globally coherent.”

Apart from confirming that classical optics and coherence theory give identical results for incoherent sources such as assumed here, this example also points to a communications gap between laboratory physicists and coherence theorists. The word “diffraction” is not mentioned once in the Kandpal, Vaisha and Joshi and Foley papers. It is hoped that the above discussion will help bridge this gap.

### 5.3 Example: Spectral Changes Produced in Young’s Interference Experiment

**Fig. 5:**
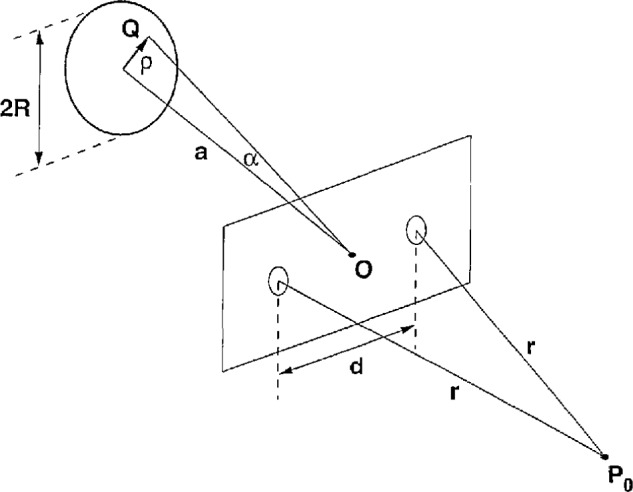
Young’s interference experiment.

In a paper referenced above, James and Wolf [13] considered the optical setup depicted in Fig. 5, in which an incoherent, plane, polychromatic source with angular radius *α=R/a* is used to illuminate a screen containing two circular pinholes of area *A* which are located at distances *±d*/2 from the source normal. The source and pinhole planes are parallel to one another, are separated by a distance which is large enough to justify the Fraunhofer approximation, and the interference effect is observed at a similarly large distance from the aperture. The point of observation *P*_0_ is located on axis and at equal distances *r* from the two pinholes, so that “colour effects, which are usually produced in interference experiments with broadband light, are absent.” James and Wolf found the following expression for the spectrum observed at *P*_0_,

$$S\left(P_{0},\omega \right)=2S^{\left(i\right)}\left(\omega \right){\left(A/2\pi cr\right)}^{2}\omega^{2}[1+2J_{1}\left(\omega \alpha d/c\right)/\left(\omega \alpha d/c\right)].$$ (24)

The conclusion of their paper was that the spectral effects produced by the experiment are two-fold: a blueshift due to diffraction which is described by the factor *ω*^2^ in Eq. (24), and a redshift due to the finite size of the source which is described by the Bessel function in the same equation.

James and Wolf expected an achromatic fringe in the center of the interference pattern. Quite the opposite is true. The central blueshift due to diffraction is to be expected for the same reason as previously illustrated in Fig. 4. It represents a classical phenomenon which is described by the 1/λ^2^ scale factor of the Huyghens-Fresnel-Kirchhoff diffraction formula Eq. (21).

The central redshift due to the finite size of the source can likewise be explained by classical reasoning. In the notation of Fig. 5, the contribution of a source point *Q* to the interference pattern at *P*_0_ is given by [1 + cos(*ω∆*/*c*)] d***Q***, where *∆* = (*QS*_2_ + *S*_2_*P*_0_) *−* (*QS*_1_ + *S*_1_*P*_0_) = *QS*_2_ − *QS*_1_ is the path difference of the interfering light. Referred to polar coordinates with the origin at the center *O* of the pinhole screen, we have ***Q*** = (*ρ* cos*χ*,*ρ* sin*χ*, −*a*), *S*_1,2_ = (±*d*/2, *O*, *O*), (*QS*_1_)^2^ − (*QS*_2_)^2^ = 2*ρd* cos*χ*, *QS*_1_ + *QS*_2_~2*a*, so that

$$\Delta =\left[{\left(QS_{1}\right)}^{2}-{\left(QS_{2}\right)}^{2}\right]/\left(QS_{1}+QS_{2}\right)∼\left(\rho d/a\right)\cos\chi .$$ (25)

Hence, the spectral radiant flux produced at *P*_0_ by the source as a whole will be proportional to

$$\begin{array}{c} \int \rho d\rho \int d\chi \left\{1+\cos\left[\left(\omega \rho d/c\right)\cos\chi \right]\right\}\\ =2\pi \int \rho d\rho \left[1+J_{0}\left(\omega \rho d/ac\right)\right]\\ =\pi R^{2}\left[1+2J_{1}\left(\omega \alpha d/c\right)/\left(\omega \alpha d/c\right)\right], \end{array}$$ (26)

which is in agreement with Eq. (24). The physical interpretation of the redshift is that each point of the primary source produces its own interference pattern, the width of the fringes increasing with wavelength so that the outer portions of the source contribute more red than blue light to the central fringe.

In a subsequent paper [14], James and Wolf considered the off-axial spectral effects produced by Young’s experiment in a paraxial approximation. They obtained, instead of Eq. (24),

$$\begin{array}{c} S\left(x,\omega \right)=2S^{\left(i\right)}\left(\omega \right){\left(A/2\pi cr\right)}^{2}\omega^{2}\\ \times \left\{1+2\left|J_{1}\left(\omega \alpha d/c\right)/\left(\omega \alpha d/c\right)\right|\cos\left(\beta +\omega xd/rc\right)\right\}, \end{array}$$ (27)

where *x* is the lateral distance of the point of observation from the central point *P*_0_, and *ß* = 0 or π according to whether *J*_i_(…) is positive or negative. James and Wolf stated that they were dealing with “new aspects” of Young’s experiment.

Since an incoherent primary source was assumed, the spectral modifications described by Eq. (24) are classical effects. They were first investigated by Fraunhofer [1] and are explained in textbooks, including those by Jenkins and White [36] and Strong [37]. Briefly stated, although white-light interference may not be visible to the unassisted eye, it still takes place in Young’s experiment and can be observed with a spectroscope. The spectroscope reveals “channeled spectra” in the form of dark bands in the neighborhood of wavelengths for which destructive interference takes place, and enhanced colors near wavelengths that interfere constructively. This is shown in the various figures of Ref. [14], and also by colored drawings in the 1897 edition of Müller-Pouillet’s “Lehrbuch” [38]. Nonetheless, the elucidation of channeled spectra by coherence theory is interesting and offers a novel point of view.

## 6. Concluding Remarks

We have attempted to reconcile conflicting points of view of laboratory physicists and coherence theorists on correlation-induced spectral changes arising from the partial coherence of primary and secondary light sources. We have shown that, under normal laboratory conditions and in the Fraunhofer approximation, the directional spectrum of light does not change on propagation in free space and that the total spectrum is preserved in accordance with the principle of energy conservation. We have demonstrated that descriptions of spectral phenomena by the theory of partial coherence and by classical wave optics theory are fully equivalent for incoherent primary sources. We have emphasized that a statistical approach is essential, and coherence theory is required, for partially coherent primary sources.

We have also pointed out that the theoretical description of light propagation in the proximity of sources is, by and large, still an unsolved problem. Most publications on partial coherence (this one included) use the Fraunhofer approximation, whereas optical radiometry often requires at least the Fresnel approximation. Theorists could make valuable contributions to optical radiometry by turning to this issue.

The essence of Wolf’s original paper [2] on correlation-induced spectral changes should not be misconstrued as a contradiction or modification of the classical wave theory of incoherent physical sources. Rather, it should be regarded as a generalization and extension of classical optics that allows the description of phenomena arising from the properties of existing or newly developed partially coherent sources. Wolf’s formulation of the conditions under which the spectrum of a partially coherent source is propagated isotropically may lead to the development of new imaging devices that could reduce the magnitude of diffraction errors in optical radiometry.
